# Determinants of daily smoking in Turkish young adults in the Netherlands

**DOI:** 10.1186/1471-2458-6-294

**Published:** 2006-12-06

**Authors:** Floor VA van Oort, Jan van der Ende, Alfons AM Crijnen, Frank C Verhulst, Johan P Mackenbach, Inez MA Joung

**Affiliations:** 1 Department of Public Health, Erasmus MC, University Medical Center, Rotterdam, the Netherlands; 2 Department of Child & Adolescent Psychiatry, Erasmus MC-Sophia, University Medical Center, Rotterdam, the Netherlands

## Abstract

**Background:**

As little is known about the determinants of smoking in large ethnic minorities in the Netherlands and other Western European countries, we studied the determinants of smoking young adult offspring of Turkish migrants to the Netherlands.

**Methods:**

Cross-sectional survey of 439 Turkish adults (18–28 y) in 2003. Smokers were compared with never smokers for five groups of determinants: demographic and socioeconomic factors, behavioral and emotional problems, psychosocial factors, and cultural factors. Associations were measured by prevalence rate ratios.

**Results:**

Prevalences for men were 51% for daily smoking, 12% for former smoking, and 38% for never smoking. For women they were 44%, 11%, and 47%, respectively. Without adjustment for other determinants, higher prevalence was associated with: emotional problems, boredom, life events, and being male; and, specifically among women, with low self-esteem and having children. The strongest determinants of daily smoking In multivariate models were alcohol use and demographic and socio-economic factors. Of the cultural factors only strong Muslim identification was associated with lower smoking prevalence.

**Conclusion:**

The high prevalence of smoking warrants action. Many of the well-known determinants of smoking in Western countries were also important among young adults from ethnic minorities. Women with children and people of a low educational level deserve special attention.

## Background

Smoking is one of the greatest public health concerns [1,2], on which many policies and health promotion campaigns have been implemented, especially over the past two decades. Although it is a modifiable risk factor, effective interventions and the identification of priority groups require fuller understanding of the determinants of smoking behavior.

These determinants have been explored in many different domains. For example, smoking prevalence has been found to be higher among the following groups: men (e.g. [3]), the lower educated in Western countries (e.g. [4,5]), the unemployed (e.g. [6]), individuals suffering adverse life events or chronic stress (e.g. [5,7]), those with low self-esteem (e.g. [7]), those with little control over their lives (e.g. [7,8]), those who are involved in other types of risk behavior (e.g. [5,9]), and those with emotional problems (e.g. [7,10,11]). In certain cases, determinants were sex specific (e.g. [6,12]).

While the prevalence of smoking is high in several migrant populations [13-15], few studies have been conducted among ethnic minorities and migrants. Most of these studies were done in the UK and USA. Although most show that the determinants of smoking in migrants and their offspring are similar to those of the majority population [16-19], associations of smoking with socio-economic position were mixed [18,20,21]. Among migrants there may also be culture-specific determinants of smoking, such as discrimination and ethnic identity (e.g. [20,22-25]). Acculturation theories predict that migrants will eventually adopt the behaviors of the host population they come into contact with [26,27]. In addition, the association between acculturation and smoking may vary by educational level of migrants [28,29].

With regard to large ethnic minorities in Western European countries other than the UK and the USA knowledge of the question is limited [28,29]. With over 3 million members, Turks are now the largest immigrant group in the European community, Turkish immigrants having arrived as labor migrants in countries such as the Netherlands, Belgium, Germany, France and Sweden between 1960 and 1980.

Compared to levels in Dutch natives and in migrants from other countries living in the Netherlands, smoking prevalence is high among Turkish male labor migrants (42–73%), although it is lower among females (13–34%) [28]. We tested whether the well-known determinants of smoking apply to young Turkish adults in the Netherlands. We studied five groups of determinants: emotional and behavioral problems, and demographic, socioeconomic, psychosocial and cultural factors.

## Methods

### Participants

In 1993, 1198 children aged 4–18 y with one or both parents born in Turkey were randomly selected from the municipal registers in Rotterdam and The Hague [30]. Parents and children were interviewed at home. In 2003, the children -by then young adults- were interviewed again, and information about smoking behavior and determinants was collected.

Of the original 1993 sample, 132 persons were excluded because they did not fulfill inclusion criteria (n = 19), or because the address provided was incorrect (n = 113) (Table 1). Respondents and non-respondents of the original representative sample of 1993 were traced through the municipal registers.

Table 1 shows the flow of participants. Using municipal registers, it was possible to trace 994 (93%) of the 1066 children. Two had died. Individuals who had moved outside the Rotterdam and The Hague regions could not be contacted (n = 18).

For participation, we approached 974 people aged 14–28 y (91% of 1066). Four had no parents born in Turkey, and two had severe intellectual disability. All six were excluded, leaving 968 in all. The response rate was 64% (621 out of 968), with three main reasons for non-response: refusal (17%), incorrect address (6.5%), and unreachable after at least three attempts (10%). Only young adults (18–28 y) were included in the analyses (N = 437); more details of the study have been published previously [31]. All respondents were interviewed at home by a bilingual interviewer speaking Dutch. Most respondents had been born in the Netherlands (78%), those born in Turkey having arrived at an early age (median 3.5 y). Most of them understood the Dutch language very well.

The ethics committee of Erasmus University Medical Center approved the study. All participants have given written informed consent.

Attrition analyses showed that individuals who could not be approached (n = 1066-974 = 92) did not differ in sex or age from those who had been approached. Respondents were slightly younger than non-respondents (21 y vs. 22 y, *p *< 0.0001). Attrition was not selective for sex, mental health in adolescence in 1993, country of birth, or parents' socio-economic position.

### Variables

#### Smoking behavior

Respondents reported on whether they smoked, or had smoked in the past, and on how many cigarettes they smoked a day. On this bases, they were classified as daily smoker, former smoker, or never smoker. Two respondents were excluded from the analyses because information on smoking behavior was missing, or because the respondent smoked occasionally (but not daily).

Categories of the determinants described below are displayed in table 2.

#### Demographic factors

Demographic factors comprised age, sex, living with partner, and living with own children. Living with partner was defined as being married or having cohabitated for at least six months.

#### Socio-economic factors

Socio-economic factors were educational level and number of spells of unemployment after leaving school. Current education was categorized as low (drop-out, lower vocational training), moderate (intermediate vocational training), and high (higher vocational or academic training).

#### Emotional and behavioral problems

Emotional and behavioral problems were alcohol use, externalizing problems (i.e., aggressive, delinquent and intrusive behaviors), and internalizing problems (i.e., anxiety/depression, psychosomatic complaints, and being withdrawn). These problems were measured using the Adult Self-Report [32]. The Externalizing Problems Scale consists of 36 items (Cronbach's alpha 0.88), and the Internalizing Problems Scale of 39 items (Cronbach's alpha 0.91). Categories were formed with the eightieth-percentile of the distribution as cut-off value.

#### Psychosocial factors

Psychosocial factors included boredom, number of life events experienced in the previous year, locus of control [33], and self-esteem [34]. As well as house-breaking or fire, the life events were death, an accident, problems with the law, financial problems, divorce, and health problems of a family member. For locus of control we summed the seven items (Cronbach's alpha 0.73), categorizing total scores in the upper quartile of the distribution as internal locus of control and those in the lowest quartile as external locus of control. Self-esteem (10 items, Cronbach's alpha 0.84) was similarly categorized with scores in the upper quartile labeled as high and those in the lowest quartile as low.

#### Cultural factors

Cultural factors were discrimination, having Dutch friends, ethnic identity, Muslim identity and generation. Discrimination was measured with on the basis of one item: "Generally speaking, how often do you feel you are discriminated against because you are Turkish?". Items for ethnic and Muslim identity were rated on a scale ranging from 'totally disagree (1)' to 'totally agree (5)'. Ethnic identity was assessed on the basis of the items 'I consider myself to be Turkish' and 'I consider myself to be Dutch', and answers were dichotomized (above/below 4). We used a 5-item instrument to measure Muslim identification [35], which included cognitive Muslim identity, emotional attachment, and identification as a Muslim (Cronbach's alpha 0.81). The average score was dichotomized (above/below 4).

### Statistical analyses

We determined the proportion of smokers within each category of determinants, and tested for differences with a Chi-square test. Prevalence rate ratios were calculated as measure of relative risk (RR) [36], the relative risks expressing how much higher the prevalence of smoking is in one group than in another. For example, a prevalence that was twice as high for men than for women would thus yield a RR of 2.0. All variables from one set of predictors (e.g. demographic factors) were entered in the first series of models (models 1). For the second model (model 2) we entered all variables in one multivariate model. Interactions of each of the determinants with sex and education were assessed in the regression models. Significance was set at p < 0.05, and borderline significance at 0.05 < p < 0.10.

## Results

The prevalence of daily smoking was 47% (n = 204), of former smoking 11% (n = 50), and of never smoking 42% (n = 183). For men prevalences were 51% daily smoking, 12% former smoking, and 38% never smoking; for women they were 44%, 11%, and 47%, respectively. Because the group of former smokers was too small for separate analyses, our findings compare the daily smokers with the never smokers; when former and never smoker groups were merged, findings were similar.

Table 2 presents the distribution of determinants in the study. A quarter of the respondents had a high educational level. Only 3% had experienced a divorce. Most were born in the Netherlands (78%). The median age of arrival in the Netherlands of respondents who were born in Turkey was 3.5 y, with only 10% older than ten at arrival.

Table 3 shows the proportion of smokers by determinant categories (former smokers were excluded). Overall, the associations between smoking and the determinants were as we had expected. Smoking was more prevalent for the following: men, adults living with a partner and/or children, adults who experienced unemployment, used alcohol, had externalizing and/or internalizing problems, often felt bored, experienced multiple adverse life events, had external locus of control, or had low self-esteem.

Interestingly, smoking was more common among people with a lower educational level, a pattern that is particularly common in Western populations. Of the cultural factors, only Muslim identification was associated with smoking. Similar associations were found when determinants were adjusted for the other determinants in their group (model 1 in Table 3), with the exception of externalizing problems and locus of control. Two determinants predicted smoking among women but not among men: living with children (RR women 1.54 (95%-Confidence Interval (CI) 1.07, 2.22)), men 0.84 (95%CI:0.58, 1.23)), and low self-esteem (RR women 2.45 (95%CI:1.28, 4.69), men 1.00 (95%CI: 0.65, 1.54)).

In the fully adjusted model, the only determinants of daily smoking were living with partner, low education, and alcohol use; boredom and Muslim identification were also associated, but more weakly. Low self-esteem was a determinant for women only (RR 2.15 (95%CI: 1.19, 3.88), men 0.90 (95%CI: 0.56, 1.43)). Experience of multiple adverse life events was a determinant among the higher educated only (RR ≥ 3 events: high 3.50 (95%CI: 1.09, 11.21), low 1.09 (95%CI: 0.69, 1.72)). The relative risks of the most distal determinants (demographic and socio-economic factors) were attenuated compared with the first model. This may be because part of the association between smoking and demographic and socio-economic factors are mediated by more proximal determinants including emotional end behavioral problems and psychosocial factors.

## Discussion

In young Turkish adults, smoking was rekated with many of the well-known determinants of smoking behavior. With the exception of Muslim identity, cultural factors were not related.

The prevalence of smoking of young urban Turkish men in the Netherlands was higher than that of Dutch young men living in large cities [37], and was lower than that of first-generation Turkish male migrants [28]. For Turkish young women the smoking rate was similar to that of their Dutch peers in large cities [37], and higher than that of first-generation Turkish female migrants [28]. Compared overall with Turkish adults living in Turkey (men 51%, women 11%) [38], and with those in large cities in Turkey, young Turkish men's prevalence of smoking in the Netherlands was lower (men in Ankara 65%, and Istanbul 64%). Findings for women were mixed (women in Ankara 8%, and Istanbul 56%) [39,40]. In Istanbul, the prevalence of smoking was especially high among young adults [40].

Studies of determinants of smoking among young adults in Western countries showed similar positive associations as this study for determinants as low education [5,16,41], living with children (especially for lower socio-economic groups) [5,16,42], emotional problems and behavioral problems [5,11,16,43], and low self-esteem [16]. However, findings were different for some of the determinants. For example, previous findings were mixed with regard to differences in smoking behavior between young adults with and without a partner [16,41]. In another study, smoking behavior depended on the smoking behavior of the partner [5]. Similarly, sex differences have been found for unemployment, with a stronger association with smoking among young women than among young men [6,12]. In our study, the number of frequent unemployed may have been too small to reveal sex differences.

In line with our findings, most studies of the determinants of smoking behavior have shown similarities between ethnic groups [12,17,19,21,25,42,44,45]. Many of these studies were among adolescents, and emphasized the start of smoking and the transition from experimental smoking to regular smoking. However, as most of these studies were conducted in the US, little is known about smoking determinants across ethnic minorities in Europe.

The strongest determinant of smoking in our study was level of education. Whereas smoking in Western countries is more prevalent among those with a lower educational level [4], in developing countries there is either no association, or the association is precisely the opposite. The diffusion of innovations theory predicts that as cigarette use spreads through a population and begins to decline, socio-economic patterns of smoking shift from a concentration among higher socio-economic groups (positive gradient) to one among lower socioeconomic groups (negative gradient) [46,47]. It is still unclear how long it will take before the negative gradient in smoking found in Western populations also appears among ethnic minorities [18]. In the US and Canada, the negative gradient was more pronounced for second-generation migrants than for first-generation ones; for third-generation migrants it was even more pronounced [18,48]. Our results showed already a clear negative gradient for Turkish young adults (migrant offspring) in the Netherlands.

With the exception of Muslim identification, the cultural determinants in this study were not related with smoking prevalence. It is possible that those with a strong personal identification as Muslims are stricter in their adherenc to Islamic laws, which forbid the use of intoxicants, addictive substances, and substances harmful to health [25]. Unlike our findings, three previous studies found more smokers among black Americans who experienced discrimination [24].

One of the strengths of this study is that it is the first to report on smoking behavior and its determinants in young adult migrant offspring in continental Europe. However, while it covered a wide range of well-known determinants, some were not included, such as self-efficacy, material deprivation, attitudes towards smoking behavior and cessation, and smoking behavior of peers and family. For factors of social cognition theories, also many similarities were found among first-generation migrants in the Netherlands compared with Western populations [49]. Further, although all associations were cross-sectional and thus useful for distinguishing daily smokers from never smokers, they were unsuitable for assessing causality. Unfortunately, the group of former smokers was too small to allow for the separate analyses that would provide greater insight into the predictors of smoking cessation.

Our results nonetheless provide insight into several issues relevant to prevention. Firstly, most of the determinants were similar to those found for young adults in Western countries. This suggests that (preventive) interventions targeting populations at risk on the basis of the determinants we studied, might also be useful for young migrant Turkish adults. However, this suggestion will have to be examined further. Secondly, our finding of a strong negative socio-economic gradient for both men and women suggests 1.) that interventions to help smoking cessation should focus especially on the lower educated, and 2.) that prevention of smoking should focus especially on adolescents in lower vocational schools. Thirdly, at 64%, smoking prevalence was particularly high among Turkish women with children. The harmful effects of passive smoking for children make this of particular concern [50]. Pregnant Turkish women and young Turkish mothers should therefore be a priority group for smoking interventions. Finally, as smoking is more prevalent among Turkish young adults with a partner, it would be useful to involve partners in smoking cessation programs.

## Conclusion

This study shows many of the well-known determinants of smoking in Western countries also to be determinants in young adult migrant offspring. Prevalence of smoking was high and warrants intervention and prevention. In this respect, two groups are of special interest: adults with low educational level and women with children.

## Competing interests

The author(s) declare that they have no competing interests.

## Authors' contributions

FvO coordinated the data collection, performed statistical analyses and drafted the manuscript. JvdE contributed to the interpretation of the data and helped to draft the manuscript. AC participated in the design of the study, in the data collection and helped to draft the manuscript. FV conceived the study, contributed to the design of the study and critically revised the manuscript. JM conceived the study, contributed to the design and coordination of the study and critically revised the manuscript. IJ conceived the study, contributed to the design and coordination of the study and helped to draft the manuscript. All authors approved the final manuscript.

## Pre-publication history

The pre-publication history for this paper can be accessed here:



## Figures and Tables

**Table 1:** Flow of participants

**1993**	**1198**	**Random sample (4–18 y)**		
			19	Did not fulfill inclusion criteria
			113	Incorrect address provided
	1066	Eligible		
**2003**	**1066**	**Target sample (14–28 y)**		
			**Loss to follow-up (9% of 1066)**	
			72	Tracing unsuccessful
			2	Deceased
			18	Moved out of region
	974	Approached (91% of 1066)		
			6	Did not meet inclusion criteria
	968	Eligible	**Non-response**	
			168	Refusal
			97	Unreachable ^a^
			64	Incorrect address
			18	Other reasons
	**621**	**Respondents (14–28 y) (64% of 968)**	184	14–17 years old
	**437**	**Respondents (18–28 y)**		

^a ^Unreachable after at least 3 attempts at different times over different days

**Table 2:** Distribution of determinants

	**N**	**(%)**		**N**	**(%)**
**Demographic factors**			**Psychosocial factors**		
Sex			Feel bored		
Women	189	49	Sometimes/never	316	82
Men	198	51	Often	71	18
Age			Life events		
18–23 y	257	66	0	139	36
24–28 y	130	34	1–2	201	52
Living with partner			≥ 3	47	12
No	265	68	Locus of control		
Yes	122	32	Internal	93	24
Living with children			Neutral	203	52
No	321	83	External	91	24
Yes	66	17	Self-esteem		
**Socioeconomic factors**			High	102	26
Education			Medium	196	51
High	93	24	Low	89	23
Middle	169	44	**Cultural factors**		
Low	125	32	Discrimination		
Unemployment			Sometimes/never	318	84
No spells	281	73	Often	61	16
1 spell	81	21	Missing	8	
≥ 2 spells	25	6	Dutch friends		
**Emotional and behavioral problems**			No	69	18
Alcohol use			Yes	318	82
No	295	76	Dutch identity		
Yes	92	24	No	313	81
Externalizing problems			Yes	74	19
No	312	81	Turkish identity		
Yes	75	19	No	49	13
Internalizing problems			Yes	338	87
No	317	82	Muslim identity		
Yes	70	18	No	128	33
			Yes	259	67
			Generation		
			Second	300	78
			First	87	22

N = 387, 50 former smokers were excluded

**Table 3:** Relative risks for smokers compared with never smokers by determinants

	**Smoker**	**Model 1**	**Model 2**
			
	**(%)**	**RR (95% CI)**	**RR (95% CI)**
**Demographic factors (N = 387)**			
Age			
18–23 y	50	-	-
24–28 y	57	0.94 (0.75, 1.18)	1.01 (0.81, 1.26)
Sex			
Women	47*	-	-
Men	58	1.28 (1.06, 1.55)*	1.15 (0.94, 1.40)
Living with partner			
No	47**	-	-
Yes	65	1.35 (1.07, 1.71)*	1.23 (1.01, 1.52)*
Living with children			
No	49*	-^a^	-
Yes	68	1.19 (0.91, 1.55)	1.11 (0.87, 1.42)
**Socioeconomic factors (N = 387)**			
Education			
High	35**	-	-
Middle	49	1.35 (0.98, 1.84)†	1.30 (0.95, 1.78)†
Low	70	1.81 (1.33, 2.47)**	1.59 (1.15, 2.19)*
Unemployment			
No spells	47*	-	-
1 spell	62	1.15 (0.93, 1.42)	1.03 (0.83, 1.27)
≥ 2 spells	80	1.39 (1.09, 1.78)*	1.23 (0.90, 1.67)
**Emotional and behavioral problems (N = 387)**			
Alcohol use			
No	45**	-	-
Yes	77	1.68 (1.42, 2.00)**	1.46 (1.20, 1.79)**
Externalizing problems			
No	49*	-	-
Yes	65	1.04 (0.83, 1.31)	1.15 (0.91, 1.45)
Internalizing problems			
No	50*	-	-
Yes	66	1.24 (1.00, 1.55)*	0.89 (0.70, 1.14)
**Psychosocial factors (N = 387)**			
Feel bored			
Sometimes/never	48**	-	-
Often	70	1.31 (1.09, 1.59)*	1.19 (0.98, 1.44)†
Life events			
0	45**	-	-^b^
1–2	54	1.16 (0.93, 1.45)	1.10 (0.87, 1.39)
≥ 3	68	1.38 (1.05, 1.81)**	1.25 (0.93, 1.70)
Locus of control			
Internal	44**	-	-
Neutral	48	0.88 (0.64, 1.21)	0.89 (0.66, 1.20)
External	71	1.16 (0.82, 1.64)	1.11 (0.78, 1.57)
Self-esteem			
High	40**	-^a^	-^a^
Medium	52	1.28 (0.93, 1.76)	1.11 (0.82, 1.50)
Low	67	1.43 (0.99, 2.05)†	1.23 (0.86, 1.76)
**Cultural factors (N = 379)**			
Discrimination			
Sometimes/never	52	-	-
Often	56	1.05 (0.81, 1.36)	0.93 (0.71, 1.22)
Dutch friends			
No	57	-	-
Yes	52	0.88 (0.69, 1.12)	1.00 (0.78, 1.27)
Dutch identity			
No	51	-	-
Yes	57	1.12 (0.88, 1.41)	1.21 (0.96, 1.52)
Turkish identity			
No	55	-	-
Yes	52	1.04 (0.79, 1.36)	1.14 (0.88, 1.48)
Muslim identity			
No	62*	-	-
Yes	48	0.76 (0.63, 0.92)*	0.83 (0.68, 1.01)†
Generation			
Second	54	-	-
First	46	0.87 (0.68, 1.12)	0.84 (0.65, 1.08)

† *p *< 0.10; * *p *< 0.05; **p < 0.001; – reference group; 50 former smokers were excluded

Prevalences were tested by Chi-square test; RR relative risk (i.e. prevalence rate ratios); 95% CI 95%-confidence interval

Model 1 determinants by group of determinants; Model 2 all determinants (n = 379)

^a ^interaction by sex *p *< 0.05. ^b ^interaction by educational level *p *< 0.05.

## References

[B1] Pierce JP (1989). International comparisons of trends in cigarette smoking prevalence. Am J Public Health.

[B2] Murray CJ, Lopez AD (1997). Global mortality, disability, and the contribution of risk factors: Global Burden of Disease Study. Lancet.

[B3] Giskes K, Kunst AE, Benach J, Borrell C, Costa G, Dahl E, Dalstra JA, Federico B, Helmert U, Judge K, Lahelma E, Moussa K, Ostergren PO, Platt S, Prattala R, Rasmussen NK, Mackenbach JP (2005). Trends in smoking behaviour between 1985 and 2000 in nine European countries by education. J Epidemiol Community Health.

[B4] Huisman M, Kunst AE, Mackenbach JP (2005). Educational inequalities in smoking among men and women aged 16 years and older in 11 European countries. Tob Control.

[B5] Tucker JS, Ellickson PL, Klein DJ (2003). Predictors of the transition to regular smoking during adolescence and young adulthood. J Adolesc Health.

[B6] Reine I, Novo M, Hammarstrom A (2004). Does the association between ill health and unemployment differ between young people and adults? Results from a 14-year follow-up study with a focus on psychological health and smoking. Public Health.

[B7] Tyas SL, Pederson LL (1998). Psychosocial factors related to adolescent smoking: a critical review of the literature. Tob Control.

[B8] Steptoe A, Wardle J (2001). Locus of control and health behaviour revisited: a multivariate analysis of young adults from 18 countries. Br J Psychol.

[B9] Laaksonen M, Prattala R, Karisto A (2001). Patterns of unhealthy behaviour in Finland. Eur J Public Health.

[B10] Breslau N, Kilbey M, Andreski P (1991). Nicotine dependence, major depression, and anxiety in young adults. Arch Gen Psychiatry.

[B11] Williams JM, Ziedonis D (2004). Addressing tobacco among individuals with a mental illness or an addiction. Addict Behav.

[B12] Weden MM, Astone NM, Bishai D (2006). Racial, ethnic, and gender differences in smoking cessation associated with employment and joblessness through young adulthood in the US. Soc Sci Med.

[B13] Bhopal R, Vettini A, Hunt S, Wiebe S, Hanna L, Amos A (2004). Review of prevalence data in, and evaluation of methods for cross cultural adaptation of, UK surveys on tobacco and alcohol in ethnic minority groups. BMJ.

[B14] Reijneveld SA (1998). Reported health, lifestyles, and use of health care of first generation immigrants in The Netherlands: do socioeconomic factors explain their adverse position?. J Epidemiol Community Health.

[B15] Lindstrom M, Sundquist J (2002). Ethnic differences in daily smoking in Malmo, Sweden: Varying influence of psychosocial and economic factors. Eur J Public Health.

[B16] Hu MC, Davies M, Kandel DB (2006). Epidemiology and correlates of daily smoking and nicotine dependence among young adults in the United States. Am J Public Health.

[B17] Griesler PC, Kandel DB, Davies M (2002). Ethnic differences in predictors of initiation and persistence of adolescent cigarette smoking in the National Longitudinal Survey of Youth. Nicotine Tob Res.

[B18] Acevedo-Garcia D, Pan J, Jun HJ, Osypuk TL, Emmons KM (2005). The effect of immigrant generation on smoking. Soc Sci Med.

[B19] Kandel DB, Kiros GE, Schaffran C, Hu MC (2004). Racial/ethnic differences in cigarette smoking initiation and progression to daily smoking: a multilevel analysis. Am J Public Health.

[B20] Perez-Stable EJ, Ramirez A, Villareal R, Talavera GA, Trapido E, Suarez L, Marti J, McAlister A (2001). Cigarette smoking behavior among US Latino men and women from different countries of origin. Am J Public Health.

[B21] Ma GX, Shive S, Tan Y, Toubbeh J (2002). Prevalence and predictors of tobacco use among Asian Americans in the Delaware Valley region. Am J Public Health.

[B22] Salant T, Lauderdale DS (2003). Measuring culture: a critical review of acculturation and health in Asian immigrant populations. Soc Sci Med.

[B23] Song YJ, Hofstetter CR, Hovell MF, Paik HY, Park HR, Lee J, Irvin V (2004). Acculturation and health risk behaviors among Californians of Korean descent. Prev Med.

[B24] Williams DR, Neighbors HW, Jackson JS (2003). Racial/Ethnic Discrimination and Health: Findings From Community Studies. Am J Public Health.

[B25] Bush J, White M, Kai J, Rankin J, Bhopal R (2003). Understanding influences on smoking in Bangladeshi and Pakistani adults: community based, qualitative study. BMJ.

[B26] Berry JW, Poortinga YH, Segall MH, Dasen PR (1992). Cross-cultural psychology: Research and applications.

[B27] Landrine H, Klonoff EA (2004). Culture Change and Ethnic-Minority Health Behavior: An Operant Theory of Acculturation. J Behav Med.

[B28] Uitewaal PJM, Manna DR, Bruijnzeels MA, Hoes AW, Thomas S (2004). Prevalence of type 2 diabetes mellitus, other cardiovascular risk factors, and cardiovascular disease in Turkish and Moroccan immigrants in North West Europe: a systematic review. Prev Med.

[B29] Nierkens V (2006). Smoking in a multicultural society: implications for prevention. PhD Thesis.

[B30] Bengi-Arslan L, Verhulst FC, van der Ende J, Erol N (1997). Understanding childhood (problem) behaviors from a cultural perspective: comparison of problem behaviors and competencies in Turkish immigrant, Turkish and Dutch children. Soc Psychiatry Psychiatr Epidemiol.

[B31] van Oort FVA, Joung IMA, van der Ende J, Mackenbach JP, Verhulst FC, Crijnen AAM (2006). Internalising and externalising behaviours in young adults: Dutch natives and Turkish migrants in the Netherlands. Ethn Health.

[B32] Achenbach TM, Rescorla LA (2003). Manual for the ASEBA Adult Forms & Profiles.

[B33] Pearlin LI, Lieberman MA, Menaghan EG, Mullan JT (1981). The stress process. J Health Soc Behav.

[B34] Rosenberg M (1989). Society and the Adolescent Self-Image.

[B35] Phalet K, van Lotringen C, Entzinger H (2000). Islam in the multicultural society [Islam in de Multiculturele samenleving].

[B36] Spiegelman D, Hertzmark E (2005). Easy SAS calculations for risk or prevalence ratios and differences. Am J Epidemiol.

[B37] van Leest LATM, van Dis SJ, Verschuren WMM (2002). Cardiovascular diseases in non-Western immigrants in the Netherlands. An exploratory study into lifestyle, risk factors, morbidity and mortality [Hart- en vaatziekten bij allochtonen in Nederland. Een cijfermatige verkenning naar leefstijl en risicofactoren].

[B38] Satman I, Yilmaz T, Sengul A, Salman S, Salman F, Uygur S, Bastar I, Tutuncu Y, Sargin M, Dinccag N, Karsidag K, Kalaca S, Ozcan C, King H (2002). Population-Based Study of Diabetes and Risk Characteristics in Turkey: Results of the Turkish Diabetes Epidemiology Study (TURDEP). Diabetes Care.

[B39] Koycu B, Kara T, Camlidag O, Aydinli R, Verschuren WM, van Montfrans GA (1997). Risk factors for cardiovascular diseases in Turks in Amsterdam and in Ankara [Risicofactoren voor hart-en vaatziekten bij Turken in Amsterdam en in Ankara]. Ned Tijdschr Geneesk.

[B40] Ogel K, Tamar D, Ozmen E, Aker T, Sagduyu A, Boratav C, Liman O (2003). Prevalence of Cigarette Use in Istanbul [Istanbul Ornekleminde Sigara Kullanim Yayginligi]. Bagimlik Dergisi.

[B41] Graham H, Francis B, Inskip HM, Harman J, SWS Study Group (2006). Socioeconomic lifecourse influences on women's smoking status in early adulthood. J Epidemiol Community Health.

[B42] Jun HJ, Subramanian SV, Gortmaker S, Kawachi I (2004). Socioeconomic disadvantage, parenting responsibility, and women's smoking in the United States. Am J Public Health.

[B43] Breslau N, Novak SP, Kessler RC (2004). Psychiatric disorders and stages of smoking. Biol Psychiatry.

[B44] Nichols TR, Birnbaum AS, Birnel S, Botvin GJ (2006). Perceived smoking environment and smoking initiation among multi-ethnic urban girls. J Adolesc Health.

[B45] Gritz ER, Prokhorov AV, Hudmon KS, Chamberlain RM, Taylor WC, DiClemente CC, Johnston DA, Hu S, Jones LA, Jones MM, Rosenblum CK, Ayars CL, Amos CI (1998). Cigarette smoking in a multiethnic population of youth: methods and baseline findings. Prev Med.

[B46] Lopez AD, Collishaw NE, Piha T (1994). A descriptive model of the cigarette epidemic in developed countries. Tob Control.

[B47] Rogers E, Schoemaker F (1971). Communication of innovations: A cross-cultural approach.

[B48] Georgiades K, Boyle MH, Duku E, Racine Y (2006). Tobacco use among immigrant and nonimmigrant adolescents: individual and family level influences. J Adolesc Health.

[B49] Nierkens V, Stronks K, van Oel CJ, de Vries H (2005). Beliefs of Turkish and Moroccan immigrants in The Netherlands about smoking cessation: implications for prevention. Health Educ Res.

[B50] DiFranza JR, Aligne CA, Weitzman M (2004). Prenatal and Postnatal Environmental Tobacco Smoke Exposure and Children's Health. Pediatrics.

